# Increased fitness of a key appendicularian zooplankton species under warmer, acidified seawater conditions

**DOI:** 10.1371/journal.pone.0190625

**Published:** 2018-01-03

**Authors:** Jean-Marie Bouquet, Christofer Troedsson, Aliona Novac, Magnus Reeve, Anna K. Lechtenbörger, Wendy Massart, Katrine S. Skaar, Anne Aasjord, Sam Dupont, Eric M. Thompson

**Affiliations:** 1 Department of Biology, University of Bergen, Bergen, Norway; 2 Sars International Centre for Marine Molecular Biology, University of Bergen, Bergen, Norway; 3 Uni Research Environment, Uni Research AS, Bergen, Norway; 4 Faculty of Science, Copenhagen University, Copenhagen, Denmark; 5 Helmholtz Centre for Ocean Research Kiel (GEOMAR), Kiel University, Kiel, Germany; 6 Department of Biology Catholic University of Leuven, Leuven, Belgium; 7 Department of Biological & Environmental Sciences, University of Gothenburg, Gothenburg, Sweden; Stockholm University, SWEDEN

## Abstract

Ocean warming and acidification (OA) may alter the fitness of species in marine pelagic ecosystems through community effects or direct physiological impacts. We used the zooplanktonic appendicularian, *Oikopleura dioica*, to assess temperature and pH effects at mesocosm and microcosm scales. In mesocosms, both OA and warming positively impacted *O*. *dioica* abundance over successive generations. In microcosms, the positive impact of OA, was observed to result from increased fecundity. In contrast, increased pH, observed for example during phytoplankton blooms, reduced fecundity. Oocyte fertility and juvenile development were equivalent under all pH conditions, indicating that the positive effect of lower pH on *O*. *dioica* abundance was principally due to increased egg number. This effect was influenced by food quantity and quality, supporting possible improved digestion and assimilation at lowered pH. Higher temperature resulted in more rapid growth, faster maturation and earlier reproduction. Thus, increased temperature and reduced pH had significant positive impacts on *O*. *dioica* fitness through increased fecundity and shortened generation time, suggesting that predicted future ocean conditions may favour this zooplankton species.

## Introduction

Through fossil fuel consumption, cement production and deforestation, CO_2_ has accumulated in the atmosphere (CO_2atm_): pCO_2atm_ estimated at 280 ppm prior to 1750, currently exceeds 400 ppm [1, 2]. This surpasses levels over the past 800,000 years, and has occurred at an unprecedented rate over the last 200 years [3]. The last time CO_2(atm)_ levels may have been comparable to current levels was between 2 and 4.6 million years ago [4] and levels are now higher than anything modern humans have previously experienced. Since the industrial revolution, approximately one third of carbon added to the atmosphere has been absorbed by the oceans [5]. Marine ecosystems are subject to combined effects imposed by climate change and other activities such as overfishing, nutrient loading, and habitat modification [6]. Two threats to marine biodiversity and ecosystem performance are warming and ocean acidification (OA). Projections indicate that by 2100, there will be a drop of 0.4 units in average surface ocean pH and a 3°C increase in temperature [7]. Present, rapid, changes generate uncertainty concerning impacts on species, adaptive capacities of natural communities, and resilience of already vulnerable marine ecosystems. Possible impacts on geochemical and biological processes, including photosynthesis, nutrient and carbon cycling, which are vital to marine ecosystems, have stimulated research into effects of these stressors on marine organisms [8]. Meta-analyses reveal variability in responses among taxa, species within taxa, populations within species, and individuals in given experiments [9]. Climate alterations can affect seasonal timing and production cycles in plankton and fish, and some major components in marine food webs are vulnerable to OA. Changes in primary production pathways may affect ecosystems by altering carbon or energy pathways and the biogeochemical quality of organic material in food webs. However, the complexity of interactions among species and differences in their sensitivities to environmental changes, make it difficult to predict the extent to which future change will disrupt ecosystems [10].

Many marine phyla contain gelatinous species (Cnidaria, Ctenophora, Tunicata, Mollusca, Chaetognatha, Annelida and Arthropoda) [11]. Gelatinous zooplankton have major roles in pelagic food webs and biogeochemical cycles, through cycling and recycling organic matter, and by influencing pelagic food web structure and elemental fluxes [12, 13]. Here, we used the appendicularian, *Oikopleura dioica*, to better understand impacts of warming and OA on a gelatinous zooplankton species, and possible consequences on pelagic food webs. *O*. *dioica* is an attractive model for cross-disciplinary research, in cell biology, chordate genetics, evolutionary-developmental biology, biomechanics, biomaterial, environmental toxicology and vertical global carbon flux [14]. Numerous genome-scale resources are available, offering interesting research perspectives in ecological and environmental genetics [15]. Next to copepods and euphausiids, appendicularians are among the more abundant components in marine mesozooplankton communities [16], acting as an important link between trophic levels and contributing to vertical carbon flux. Living inside a gelatinous house, composed of mucopolysaccharides (Oikosins) [17] and cellulose [18], appendicularians feed on small particles, including micro-phytoplankton, bacteria, and partially dissolved organic matter (DOM) down to 0.2 μm in size [19]. Coastal densities range from tens to thousands of individuals per cubic meter [20]. High nutritional quality [21], slow swimming speed, and a soft body, make appendicularians desirable prey for pelagic invertebrates (chaetognaths, copepods, ctenophores, medusa) and larval, juvenile and adult fish [22]. To maintain filtering efficiency, the house is repeatedly synthesized and discarded as the animals grow. Discarded houses, including trapped material and fecal pellets, contribute significantly to oceanic vertical carbon flux [23]. Therefore, environmental modifications impacting *O*. *dioica* could have repercussions on species interactions and carbon cycling, ranging from CO_2(atm)_ sequestration to fisheries resources. To assess responses to perturbations and understand how environmental factors drive *O*. *dioica* physiology and population dynamics, this work combined large-scale studies in pelagic ecosystems in mesocosms and targeted laboratory-scale studies. The mesocosm experiment assessed the hypothesis that predicted future ocean scenarios will favour gelatinous species in a structured ecosystem. Laboratory experiments permitted elucidation of how such physicochemical conditions impact *O*. *dioica*, providing insight into physiological keys underlying observed mesocosm responses.

A previous mesocosm experiment [24] found that projected future conditions resulted in increased *O*. *dioica* abundance, over one reproductive cycle. The phytoplankton bloom stimulated in that experiment, resulted in an increase in pH (8.40 ± 0.26) similar to that observed in productive coastal waters such as Norwegian fjords and estuaries [25, 26, 27]. Therefore, it remained unclear if lower pH positively regulated *O*. *dioica* abundance or if increased pH observed during the blooms had a negative effect. Here, a similar mesocosm experiment was conducted, but in addition to generating more acidified conditions (pH 7.6), ambient mesocosm treatments were regulated to prevent a pH increase during the algal bloom. Based on results from the two sets of mesocosm experiments, we then explored at the microcosm scale, the individual and combined effects of two temperature (Δ3°C), 3 pH (7.6, 8.0 and 8.4) and 3 diet regimes. We examined, survival, growth, fecundity and early developmental success. Our results consistently document increased fitness of *O*. *dioica* at increased temperature and reduced pH, at both mesocosm and microcosm scales.

## Materials and methods

### Mesocosms

**Fig 1:**
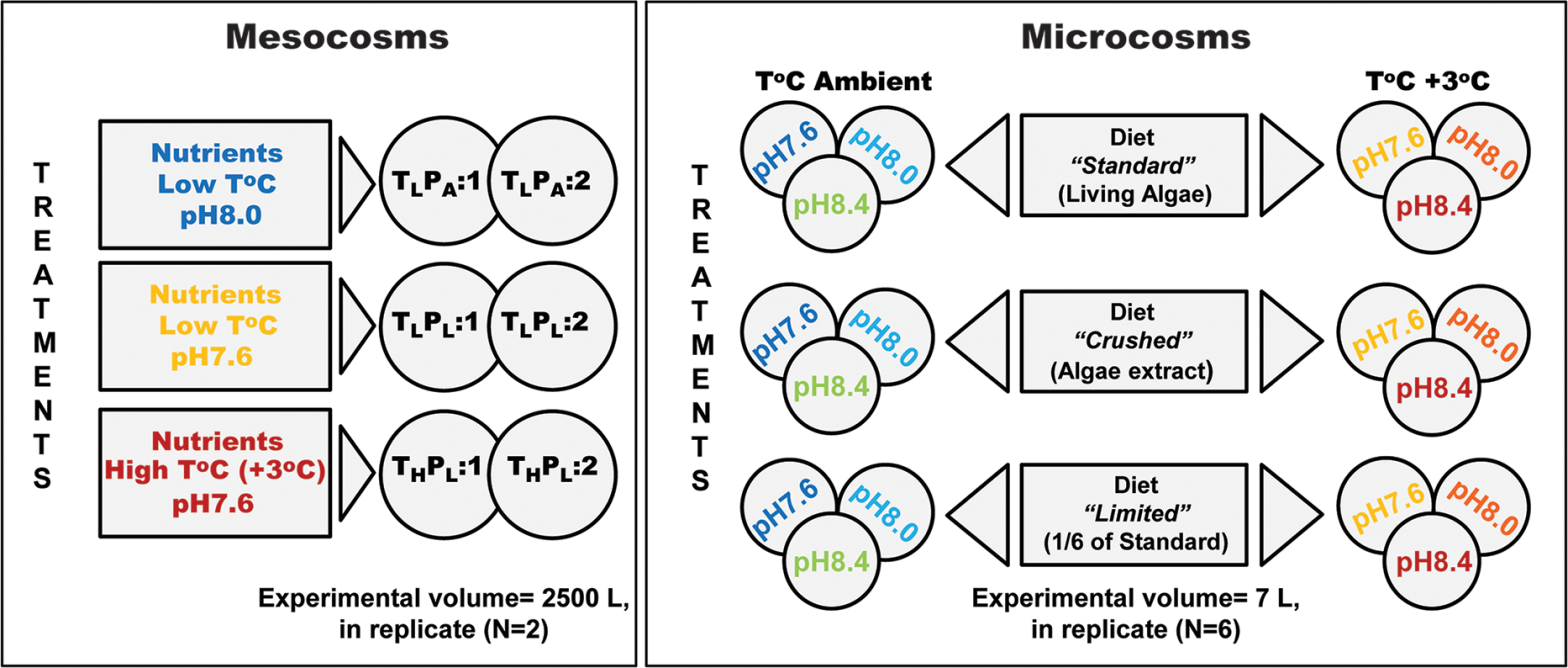
Experimental designs for mesocosm and microcosm experiments. Mesocosm data presented here are part of a larger study, but only show treatments that included *O*. *dioica*. The full mesocosm experiment included equivalent treatments, without appendicularian addition and blank treatments (fjord water ambient temperature (T_L_), ambient pH (P_A_), no nutrient addition, no *O*. *dioica* seeding) [13]. Regulation of pH was performed in all mesocosms to achieve future predicted levels (P_L_ = pH 7.6), or initial ambient fjord water levels (P_A_ = pH 8.0). This latter regulation to maintain initial ambient levels (P_A_) was done to prevent alkaline conditions resulting from the nutrient-induced algal blooms observed in previous experiments with similar design [24]. The nomenclature, TxPy:N (Temperature, pH, replicate number) used to identify each 2500 L mesocosm was as follows: T_L_, the lower temperature corresponding to the natural fjord temperature, or T_H_, higher temperature targeting a + 3°C increase relative to T_L_; P_A_, ambient initial fjord water pH, or P_L_, predicted future lower pH. Each mesocosm treatment was replicated (N = 2) and characterized as replicate: 1 and: 2, respectively. In microcosm experiments (7 L volumes) all diets were tested independently. Replicates (N = 6) were generated from 3 successive experimental runs per diet regime.

The mesocosm experiment (June 10^-^30^th^, 2011), was conducted at the University of Bergen marine station, Raunefjord, Norway. This system includes multiple 2500 L (1.5 m diameter, 1.5 m high) glass-fiber tank mesocosms open to the atmosphere. They allow studies of plankton dynamics, in complex unfiltered seawater, with targeted perturbations (S1A Fig and Fig 1) [13, 24].

On June 10^th^ (Day-1), the mesocosms were filled (300 to 500 L min^-1^), using a plankton pump (Unik Filtersystem, Oslo, Norway), with unfiltered, nutrient-poor seawater, in a staggered mode, to generate equivalent starting conditions. Most of the organisms present *in situ*, at filling, were represented with the possible exception of the gelatinous zooplankton species such as *Oikopleura dioica*, generally too sensitive to tolerate the filling process. Therefore, juvenile *O*. *dioica* (Day 1 stage) were produced in culture in sufficient amounts to seed the mesocosms, to an initial density of 10 ind.L^-1^[14, 24]. Experimental manipulations included CO_2_ enrichment, temperature increase and nutrient addition. Each mesocosm was equipped with an inverted funnel connected to an air pump to generate large air-bubbles (diameter >5 cm, every 3–5 s) for gentle mixing. A detailed description of the experimental setup, technical features and the sampling methods are described in Troedsson *et al*. [24]. Experimental seawater pH and temperature target conditions were defined after IPCC predictions for the year 2100, model RCP 8.5 corresponding to a 0.4 pH unit reduction and +3°C temperature increase relative to ambient values. The experiment included 7 treatments in duplicate for a total of 14 mesocosms. The present study focuses only on the treatments including *O*. *dioica* addition, corresponding to 3 treatments in duplicate (Fig 1).

The mesocosms were placed, in sets of 6, in larger holding tanks to regulate temperature (S1A Fig). One holding tank was equipped with a commercial heating device consisting of a 6 kW Titanium electric heating unit (Pahlén Norge AS, ref 141601T-01), a circulation pump (Astral Sena ref 25463 0,74kW, 50Hz, 230 V, mono phase, mounted on a 50 mm PVC tube) and a SAAS A/S temperature regulation system, Oslo, Norway. For this study, temperature in the relevant tanks was gradually increased during the first day to a targeted +3°C relative to fjord *in situ* temperature. All treatments, including inorganic nutrient addition (8 μM NaNO_3_, 0.5 μM K_2_HPO_4_ and 5 μM Na_2_SiO_3_), *O*. *dioica* seeding (10 ind.L^-1^), temperature and pH adjustments, were initiated on Day 0 (June 11^th^).

### Microcosms

Experiments were performed at the Sars appendicularian facility [14], with cultured populations originating from Rosslandspollen (60°34’02.1”N and 5°02’35.8”E). The setup consisted of 12 beakers (S1B Fig), each equipped with a pH probe (220.10) connected to a computerized control system (Aquamedic; 201.00). For each experimental run, beakers with different assigned pH and temperature conditions were randomly placed in the culture facility to avoid any possible biased location effects. Levels of acidification were regulated by bubbling of CO_2_ (bubble diameter ~1 mm), directly into seawater to a resolution of 0.01 pH units. Saturated lime water (Kalkwasser mix, Aquamedic) was used to achieve alkaline conditions. Fresh, saturated lime stock was prepared in milliQ water (25 g.L^-1^) and stored at 4°C. After decantation, only the clear volume was used. To control alkaline pH (3 times daily), decanted lime stock solution was diluted 50/50 in seawater prior to addition via dripping into experimental beakers.

Three diet regimes (Standard; Crushed; Limited), 2 temperatures (14±0.5°C and Δ+3±0.5°C), and 3 pH conditions (pH 7.6, 8.0 and 8.4) were used (Fig 1 and S1 Table). The “Standard” diet regime is summarized in S1 Table. The “Crushed” regime was based on extracts prepared from the 3 algal strains: *Chaetoceros calcitrans*, *Isochrysis sp*., and *Rhinomonas reticulata*. It was previously observed that extracts needed to be prepared from a three-fold increase relative to cell numbers in the standard regime in order to maintain egg production levels similar to the standard diet regime. The crushed diet was developed to obtain fecundity values in a range similar to those obtained with the standard diet, while simulating a partially “pre-digested” diet. The “Limited” diet corresponded to a dosage of one sixth of the components in the “Standard” diet [28]. The “Limited” diet results in reduced fecundity but does not introduce increased and/or variable effects on animal mortality. Thus, the standard diet tested the effects of pH and temperature when nutrition was not limiting with respect to reproductive output (e.g. encountered in bloom situations). The limited diet tested these same effects when the identical quality of nutrition was supplied but at reduced quantities. The crushed diet tested these same effects when the quantity was comparable to the standard diet (as determined by mean fecundity in preliminary runs at pH 8.0 and 15°C), but the quality was altered to a non-living “pre-digested” state. The latter two conditions are encountered in post-bloom phases. Algal culture, cell quantification and crushed extract preparations were performed as described [14]. Temperature was modified using 2 circulating water baths with heating-cooling units (TK 500; Teco®). Three experimental runs with 2 replicates were done, for a total of 6 replicates per condition. Starting animal density for each experimental run was 160 ± 10 ind.L^-1^. At the start of each experimental run, 50–55 females and 25 males were selected and placed in 6 L to spawn. From the same population, an additional 20–30 females were sampled and egg counts were made in order to determine the mean fecundity in the population. After 3 h, females that had not yet spawned were removed from the beaker and counted. The spawn was then diluted equally into each of the 12 beakers. Thus, knowing the number of spawned females, mean fecundity, and the diluted volume, we calculated the initial number of animals in each beaker at the start of the experimental run (starting density = 160 ± 10 ind.L^-1^). The number of females that successfully spawned was used as a starting reference to calculate subsequent survival rates. Spawns were diluted by gently submerging graduated propylene plastic beakers in the spawn beaker and distributing the calculated volumes to each of the experimental beakers. Stepwise temperature and pH modifications were initiated 30 min after the start of a run with targeted pH and temperature values achieved over a 6 h period. Thereafter, conditions were controlled and adjusted, if necessary, 2–3 times daily.

### Physical and chemical parameters

Temperature, pH and salinity were measured twice daily using a WTW Multi-parameter 3420 probe equipped with TetraCon® 925 (301710) conductivity and pH Sentix® 980 (103780) sensors. Calibrations were made with National Bureau of Standards (NBS) buffers (Hamilton calibration buffer). pH was regulated by discontinuous bubbling of CO_2_ gas using an AquaMedic control (AB Aqua Medic GmbH Germany) with an electronic shut-off valve for CO_2_ (M-valve standards, 230.00) that opens when pH deviates more than 0.01 above set values. CO_2_ levels were monitored using an AM-standard pH electrode (201.00), calibrated using the NBS buffer. Total Alkalinity (*A*_*T*_) was measured on filtered samples with a TitroLine Alpha Plus (SI Analytics). The *p*CO_2_ values were calculated from temperature, pH and alkalinity using CO2CALC [29], with dissociation constants [30] refitted by Dickson and Millero [31].

### Biological parameters

Abundance was monitored by counting animals in mesocosm subsamples [24]. In microcosms, daily survival was calculated based on the initial number of eggs diluted and successive animal counts. At Day 3, all animals in each beaker were counted, and a sub-fraction (210 individuals per beakers) was maintained in culture until maturation (daily transfers, counts and sampling of 20–30 individuals). Animal density was maintained at identical levels across treatments, to prevent impacts on fecundity. Animals were photographed using a binocular (Olympus SZX2-ZB16), equipped with a Nikon camera DS-5M connected to the Nikon DS-L1 on specimens sedated in 0.125 mg.mL^-1^ ethyl 3-aminobenzoate methanesulfonate salt (Sigma) (Mesocosms), or fixed in 4% paraformaldehyde, 0.1 M MOPS, 0.5 M NaCl, pH 7.5 (Microcosms). Body lengths were measured using Image Pro-plus v4.5.0.29.

Egg production was quantified in microcosms for each diet regime. Mature females were isolated (in 5 mL staining blocks) until spawning. Eggs were then photographed and counted. Generation time and maturation window were defined as previously [28]. Mature females and males were isolated and monitored until spawning to determine the time to 50% spawning, and the window for 25 to 75% of a population to have spawned. Fertilization rates and juvenile development were quantified from *in vitro* fertilizations performed at respective conditions. Embryos were incubated overnight at room temperature (19 ± 1°C) in 8 mL polypropylene tubes, on a rotating wheel (Cole-Parmer RT50; 3–5 rpm) before counting. Maximal intrinsic rates of natural increase (*r*_max_) were calculated as a measure of fitness, from the number of eggs (b) and the generation time (*T*) according to the following equation:

$$r_{max}=\frac{\ln\;b}{T}$$ (1)

### Data analyses

Statistical analyses were done using R. Data were analyzed with generalized linear mixed models (GLMM) from “MASS” [32]. Distributions used in the GLMM were quasi-poisson for counts (fecundity and abundance); quasi-binomial for percentage (survival and juvenile development), and Gaussian for size. To account for temporal autocorrelation, a first-order autoregressive term was used for repeated measurements over time. For mesocosm data, the mesocosm reference number was used as the random factor. For microcosm data at each diet regime, experimental run, replicate code (N = 6) and beaker number, were included as random factors. Main effects of experimental conditions were determined from the ANOVA outcome of the models. *Post hoc* analyses (Tukey test) used the “multcomp” package [33].

## Results

### Mesocosm experiment

#### Water characteristics

Principal physicochemical parameters measured during the 19 days of the experiment are presented in S2 Table and S2 Fig. Targeted temperature and pH values were established over 24 h, and controlled throughout the experiment, with A_T_ maintained at relatively constant levels. Initial conditions were: temperature 10.5°C, pH 8.16 (pCO_2_ = 257 μatm), and salinity 29.8. Across treatments and experimental days, mean temperatures were 12.0±0.7°C versus 14.5±0.7°C. The pH_NBS_ difference between ambient (8.02±0.10; pCO_2_ = 351±74 μatm) and acidified treatments (7.70±0.05; pCO_2_ = 814±88 μatm) corresponded to -0.32 pH units.

#### *O*. *dioica* growth and abundance

**Fig 2:**
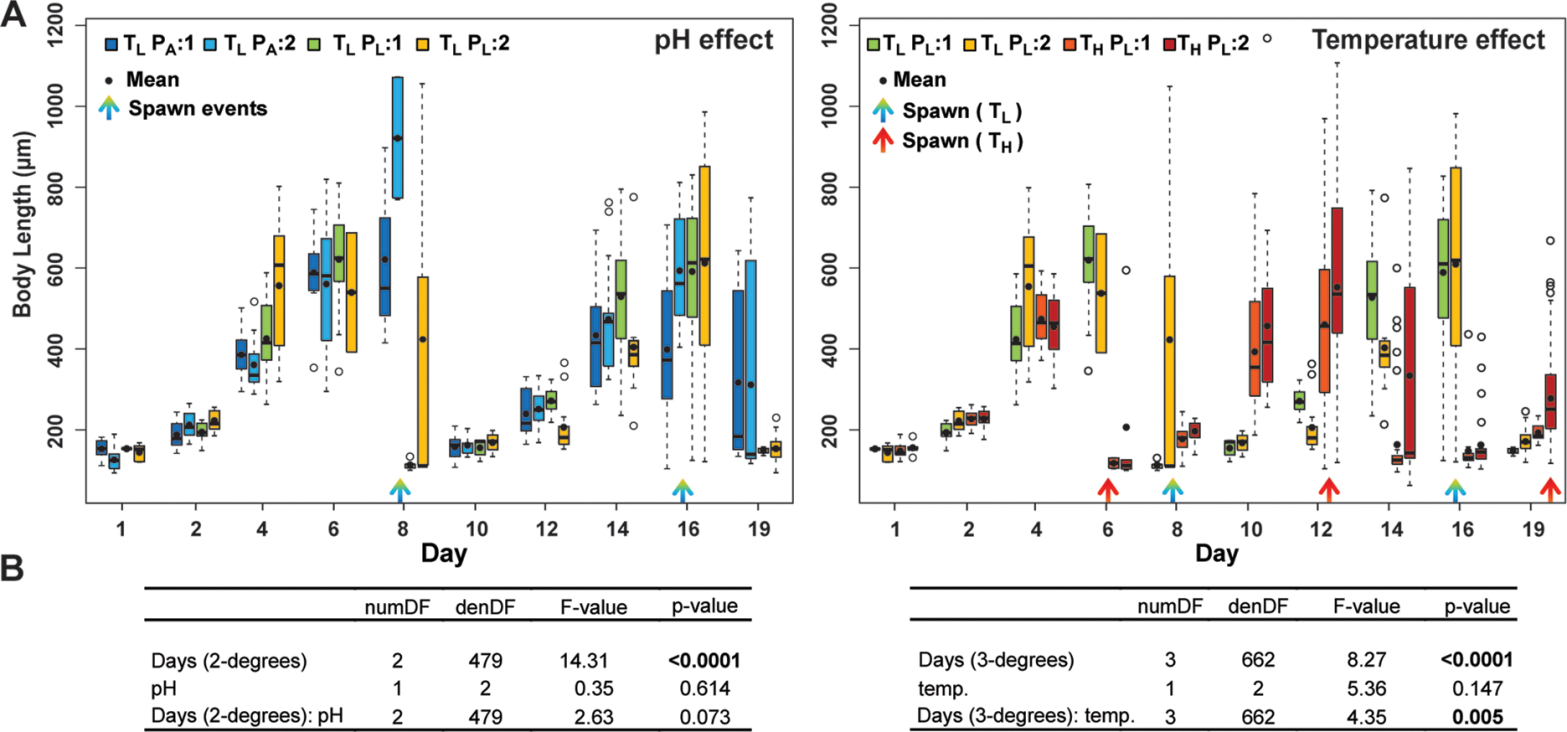
*Oikopleura dioica* growth dynamics. (A) Growth shown as a function of pH at low temperature (T_L_) (P_A_ versus P_L_) and as a function of temperature (T_L_ versus T_H_) under acidified treatment (P_L_). Growth was assessed by measuring body length. Boxplots show the median (thick horizontal black line), the lower and upper quartiles (box) and the whiskers (dashed lines) indicating maxima and minima, except when outliers were present (open circles), in which case whisker ends represent the 1.5-fold interquartile range. Means are denoted by filled circles. (B) Size variation was analyzed (ANOVA), including mesocosms (N = 2 per condition) as a random factor and a first-order autoregressive term to account for temporal autocorrelation due to repeated measurement. Polynomial regressions were fitted to the curves, with 2 degrees of polynomial in Days for pH and 3 degrees of polynomial in Days for temperature, defined as the number of spawning events observed for the respective pH and temperature analyses in (A). Significant values are indicated in bold in the respective tables (B). Treatment nomenclature: Temperature Low (T_L_); Temperature High (T_H_); pH ambient (P_A_) and pH Low (P_L_); replicate: 1 and: 2, as detailed in Fig 1.

Initially seeded at 10 ind.L^-1^ at Day0, *O*. *dioica*’s abundance decreased during the first 24 h and remained stable until the first spawning (mean across treatments 3.2±1.2 ind.L^-1^). Increased temperature accelerated growth and advanced reproduction by 1 to 2 days (Fig 2 and S3 Fig). First spawning was observed at Day6 in warm treatments (T_H_), compared to Day8 at low temperature (T_L_). During the experiment, two generations were observed at low temperature (spawns at Day 8 and 16) versus three in the warmer treatment (Days 6, 12 and 19). At the lower pH, juvenile individuals, indicative of recent spawning, appeared slightly earlier than at ambient pH (P_A_) (Fig 2A). This was indicated by the downward shift in mean body lengths at Day 8 and Day 19 in the P_L_ versus P_A_ treatments. Based on size progression, this shift resulting from pH perturbation only, was not sufficient to substantially desynchronize the population (Day 10 to 16); (Fig 2B: F_(2,479)_ = 2.63; p = 0.073). In contrast, temperature increase at lower pH accelerated developmental progression by ~2 days, sufficient for desynchronization between the populations at low and high temperature (Fig 2A), resulting in an additional spawning event (Fig 2B: F_(3,662)_ = 4.35; p = 0.005). Thus, a positive response of *O*. *dioica* populations was evident at projected future conditions (S3 Fig; F_(3,66)_ = 4.22; p = 0.009), corresponding to a 2.5-fold increase at Day7-11 (mean abundance = 25 ind.L^-1^) and 5-fold increase at Day12-15 (mean abundance = 51 ind.L^-1^) compared to initial seeding levels. On the other hand, pH decrease alone at low temperature did not significantly impact abundance (S3 Fig: F_(2,68)_ = 0.3; p = 0.774).

### Microcosm experiments

#### Seawater characteristics

Temperature, pH_NBS_ and carbonate chemistry parameters were determined over the course of the different experiments (S4 Fig), and mean values for each target condition under each diet summarized in S3 Table. Salinity ranged between 29.5 to 31.6 for all experiments and variation during any experimental run never exceeded ± 0.5 between replicates. Targeted values for pH and temperature were maintained over the entire life cycle. Mean temperature values across treatments were 14.22°C (Amb. T°C) and 17.17°C (T°C +3°C). Mean pH_NBS_ values corresponded to 7.63, 8.04 and 8.45, respectively for targeted pH 7.6, 8.0 and 8.4, corresponding to ±Δ0.41 pH units. Corresponding mean pCO_2_ values were 135.8 (pH 8.4), 389.2 (pH 8.0) and 1091 μatm (pH 7.6), respectively.

#### Survival

**Fig 3:**
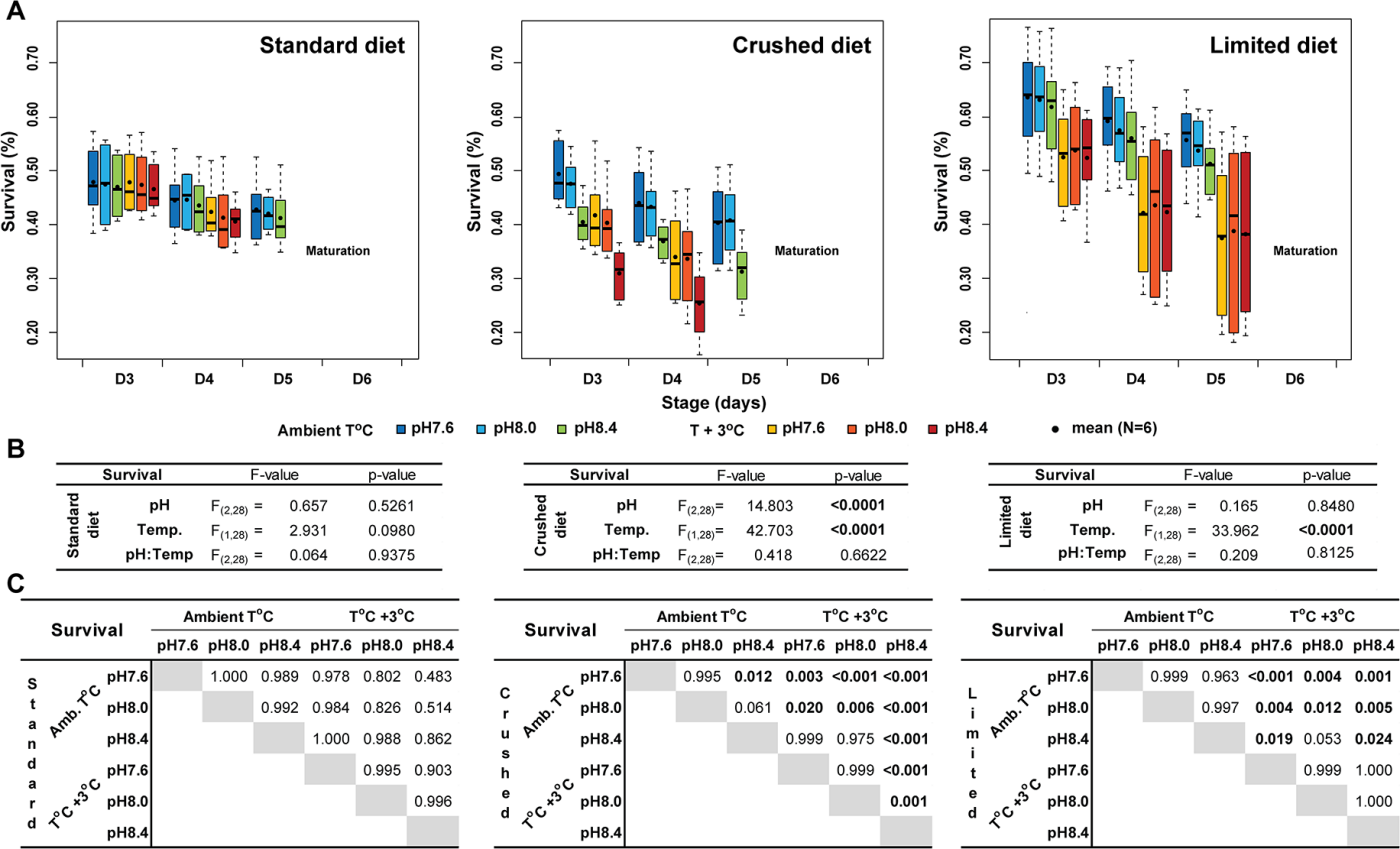
*O*. *dioica* survival in microcosms. (A) Survival from Day 3 to maturation in response to pH and temperature for each diet regime. (B), ANOVA analysis tables. (C) Tukey test results. Boxplots show the median (thick horizontal black line), the lower and upper quartiles (box) and the whiskers (dashed lines) indicating maxima and minima. When outliers are present (open circles), whisker ends represent the 1.5-fold interquartile range. Means are denoted by filled circles.

Survival was assessed from Day3 and terminated prior to spawning, when programmed mortality of this semelparous organism occurred. This corresponded to Day3-5 at ambient temperature (all diets), Day3-5 at +3°C for the limited diet, and Day3-4 at +3°C on standard and crushed diets (Fig 3). Some mortality was observed at all pH and temperature conditions, but there were significantly altered mortality levels at some of these conditions, with respect to the different food regimes. Daily mortality across the entire data set corresponded to 4.0±1.2% d^-1^ at ambient temperature (Amb. T°C) and 6.8±2.3% d^-1^ at +3°C (T°C +3°C). Under the standard diet, pH (F_(2,28)_ = 0.657; p = 0.5261) and temperature (F_(1,28)_ = 2.931; p = 0.098) had no impact on survival. At Day3, survival ranged from 46.6±4.6% at pH8.4/+3°C, to 47.9±6.7% at pH7.6/Amb. T. Just prior to sexual maturation, survival ranged from 40.6±3.9% at pH8.4/+3°C, to 42.8±6.1% at pH7.6/Amb. T. In contrast, under the crushed diet, both pH (F_(2,28)_ = 14.803; p<0.0001), and temperature (F_(1,28)_ = 42.703; p<0.0001) had significant effects. Survival levels at Day3 were equivalent at pH 7.6 and 8.0 (49.6% versus 47.7% at Amb. T and 41.9% versus 40.5% at +3°C). In contrast, survival at pH 8.4 was systematically lower (40.6% at Amb. T and 31.1% at +3°C). Multiple comparisons revealed that reduced survival in response to increased pH was significant in most cases, the only exception being at pH 8.0/Amb. T (p = 0.061). On average, increased temperature reduced survival by 9.3±1.6%, with values ranging from 7.2% (pH8.0/crushed diet) to 11.5% (pH8.4/crushed diet). For a given pH condition, increased temperature negatively impacted survival under all diet conditions. Finally, under the limited diet, survival was not impacted by pH variation (F_(2,28)_ = 0.165; p = 0.848), but was affected by temperature (F_(1,28)_ = 33.962; p<0.0001).

#### Growth

**Fig 4:**
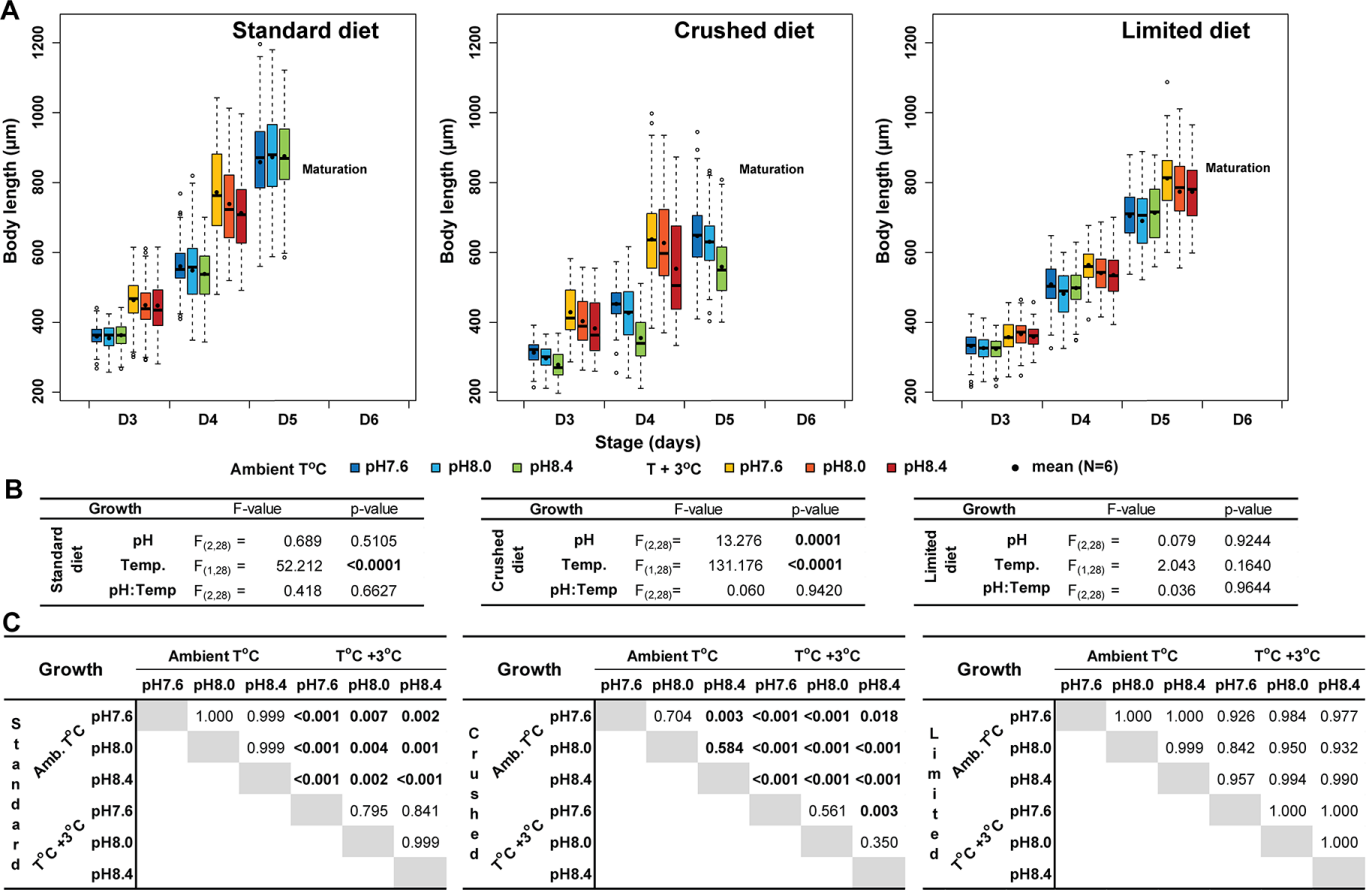
*O*. *dioica* growth in microcosms. (A) Growth from Day 3 to maturation in response to pH and temperature for each diet regime. (B) ANOVA analysis tables. (C) Tukey test results. Boxplots show the median (thick horizontal black line), the lower and upper quartiles (box) and the whiskers (dashed lines) indicating maxima and minima. When outliers are present (open circles), whisker ends represent the 1.5-fold interquartile range. Means are denoted by filled circles.

Growth from Day3 to pre-maturation was measured for each temperature and pH condition, under the respective diets (Fig 4). Under the standard diet there was no effect of pH on growth (F_(2,28)_ = 0.689; p = 0.5105) whereas temperature increase had a significant, positive impact on growth and developmental progression (maturation attained a day earlier at +3°C compared to Amb. T; (F_(1,28)_ = 52.212; p<0.0001)). Under the crushed diet, however, growth was significantly reduced at higher pH conditions (F_(2,28)_ = 13.276; p = 0.0001), with comparable effects at both ambient and elevated temperatures. Multiple comparisons revealed that this effect was significant between extreme pH conditions (pH7.6 vs pH8.4; p = 0.003 both at Amb. T and +3°C) but was not significant in comparison with ambient pH8.0 at either temperature. The mean reduction in body length between extreme pH conditions ranged between 11 and 22% as a function of developmental stage and temperature. Increased temperature alone resulted in faster growth corresponding to average increases of 37% at Day3 and 48% at Day4, across all pH conditions (F_(1,28)_ = 131.176; p<0.0001). Under the limited diet, results were similar to growth profiles under the standard diet: pH had no effect (F_(2,28)_ = 0.079; p = 0.924), but reduced food availability moderated the positive impact of increased temperature on growth, such that the temperature effect was no longer statistically significant (F_(1,28)_ = 2.043; p = 0.164). Animals were larger at +3°C, but the difference between temperatures was limited to 10% at both Day3 and 4 (mean across all pH conditions). In comparison, under the standard diet, increased temperature accelerated growth on average by 25% at Day3 and 35% at Day4.

#### Developmental and maturation kinetics

Generation time and maturation window were quantified under the standard diet (Table 1). Generation time was strongly influenced by temperature (Table 2: F_(1,17)_ = 338.6; p<0.001). The life cycle was significantly shortened by ~30 h (119–123 h at +3°C, versus 148–153 h at ambient T; Tables 1 and 3) at elevated temperature. The maturation window (Table 1) was significantly impacted by pH (Table 3: F_(2,17)_ = 13.63; p = 0.001) and temperature (Table 2: F_(1,17)_ = 13,04; p = 0.002). Increased temperature reduced the maturation window by 1–2 h (e.g.: pH7.6, 10.3 h at +3°C versus 12.0 h at Amb. T; pH8.4, 13.3 h at +3°C versus 15.5 h at Amb. T). At pH8.4, there was an increase in generation time, and a slightly expanded maturation window. Intermediate values were observed for these parameters at pH8.0, with significant differences between pH7.6 and pH8.4 (at both temperatures, Table 3).

**Table 1:** Developmental progression, reproduction and fitness comparisons between pH and temperature.

Parameters		Generation time (h)	Maturation window (h)	Fertilization rate (%)	Juvenile dev. (%)	r_max_ (d^-1^)
		mean±SE	mean±SE	mean±SE	mean±SE	mean±SE
**pH**	**Temp**.				** **	** **
pH7.6	Amb. T	148.4±2.10	12.0±0.65	96.0±0.72	67.0±2.37	0.99±0.010
pH8.0	Amb. T	149.6±2.60	12.1±0.85	96.8±0.98	67.2±2.91	0.95±0.009
pH8.4	Amb. T	153.0±1.80	15.5±0.75	96.5±0.72	65.5±2.95	0.90±0.016
pH7.6	T. +3°C	119.2±1.50	10.3±0.65	97.3±0.85	65.0±2.68	1.25±0.007
pH8.0	T. +3°C	120.6±1.75	11.0±0.55	96.9±0.45	65.4±2.37	1.18±0.019
pH8.4	T. +3°C	123.1±1.75	13.3±0.50	97.0±0.98	64.2±2.64	1.12±0.014

All experiments performed with a standard diet. h, hours; dev., development; r_max_, intrinsic rate of natural increase; SE, standard error. For Generation time and Maturation window, n = 4 beakers for each condition. For Fertilization rates and Juvenile development, n = 5 male:female matings for each condition (300 to 400 oocytes per female).

**Table 2:** ANOVA comparing impacts of pH and temperature on developmental progression, reproduction and fitness.

Parameters	Generation time	Maturation window	Fertilization rate	Juvenile dev.	r_max_
**pH**	F_(2,17)_ = 2.55	F_(2,17)_ = 13.63	F_(2,24)_ = 0.037	F_(2,24)_ = 0.169	F_(2,17)_ = 48.256
	p = 0.108	**p = 0.001**	p = 0.964	p = 0.845	**p<0.001**
**temp**	F_(1,17)_ = 338.6	F_(1,17)_ = 13.04	F_(1,24)_ = 0.837	F_(1,24)_ = 0.608	F_(1,17)_ = 664.4
	**p<0.001**	**p = 0.002**	p = 0.369	p = 0.443	**p<0.001**
**pH: temp**	F_(2,17)_ = 0.028	F_(2,17)_ = 0.062	F_(2,24)_ = 0.277	F_(2,24)_ = 0.011	F_(2,17)_ = 0.655
	p = 0.973	p = 0.940	p = 0.760	p = 0.988	p = 0.532

dev., development; r_max_, maximal intrinsic rate of natural increase. Significant values are indicated in bold.

**Table 3:** Tukey test comparing impacts of pH and temperature on developmental progression, reproduction and fitness.

Conditions	Generation time	Maturation window	Fertilization rate	Juvenile dev.	r_max_
	p-value	p-value	p-value	p-value	p-value
pH8.0—Amb. T / pH7.6—Amb. T	0.995	0.888	0.980	1.000	**0.024**
pH8.4—Amb. T/ pH7.6—Amb. T	0.376	**<0.001**	0.998	0.997	**<0.001**
pH7.6—T. +3°C / pH7.6—Amb. T	**<0.001**	0.252	0.820	0.991	**<0.001**
pH8.0—T. +3°C / pH7.6—Amb. T	**<0.001**	0.817	0.970	0.997	**<0.001**
pH8.4—T. +3°C / pH7.6—Amb. T	**<0.001**	0.570	0.946	0.962	**<0.001**
pH8.4—Amb. T/ pH8.0—Amb. T	0.714	**0.013**	1.000	0.995	**0.001**
pH7.6—T. +3°C / pH8.0—Amb. T	**<0.001**	**0.015**	0.995	0.986	**<0.001**
pH8.0—T. +3°C / pH8.0—Amb. T	**<0.001**	0.184	1.000	0.994	**<0.001**
pH8.4—T. +3°C / pH8.0—Amb. T	**<0.001**	0.993	1.000	0.948	**<0.001**
pH7.6—T. +3°C / pH8.4—Amb. T	**<0.001**	**<0.001**	0.967	1.000	**<0.001**
pH8.0—T. +3°C / pH8.4—Amb. T	**<0.001**	**<0.001**	0.999	1.000	**<0.001**
pH8.4—T. +3°C / pH8.4—Amb. T	**<0.001**	**0.070**	0.997	0.999	**<0.001**
pH8.0—T. +3°C / pH7.6—T. +3°C	0.991	0.939	0.997	1.000	**<0.001**
pH8.4—T. +3°C / pH7.6—T. +3°C	0.558	**0.002**	0.999	1.000	**<0.001**
pH8.4—T. +3°C / pH8.0—T. +3°C	0.899	**0.046**	1.000	0.999	**<0.001**

dev., development; r_max_, maximal intrinsic rate of natural increase. Significant values are indicated in bold.

#### Fecundity and egg quality

**Fig 5:**
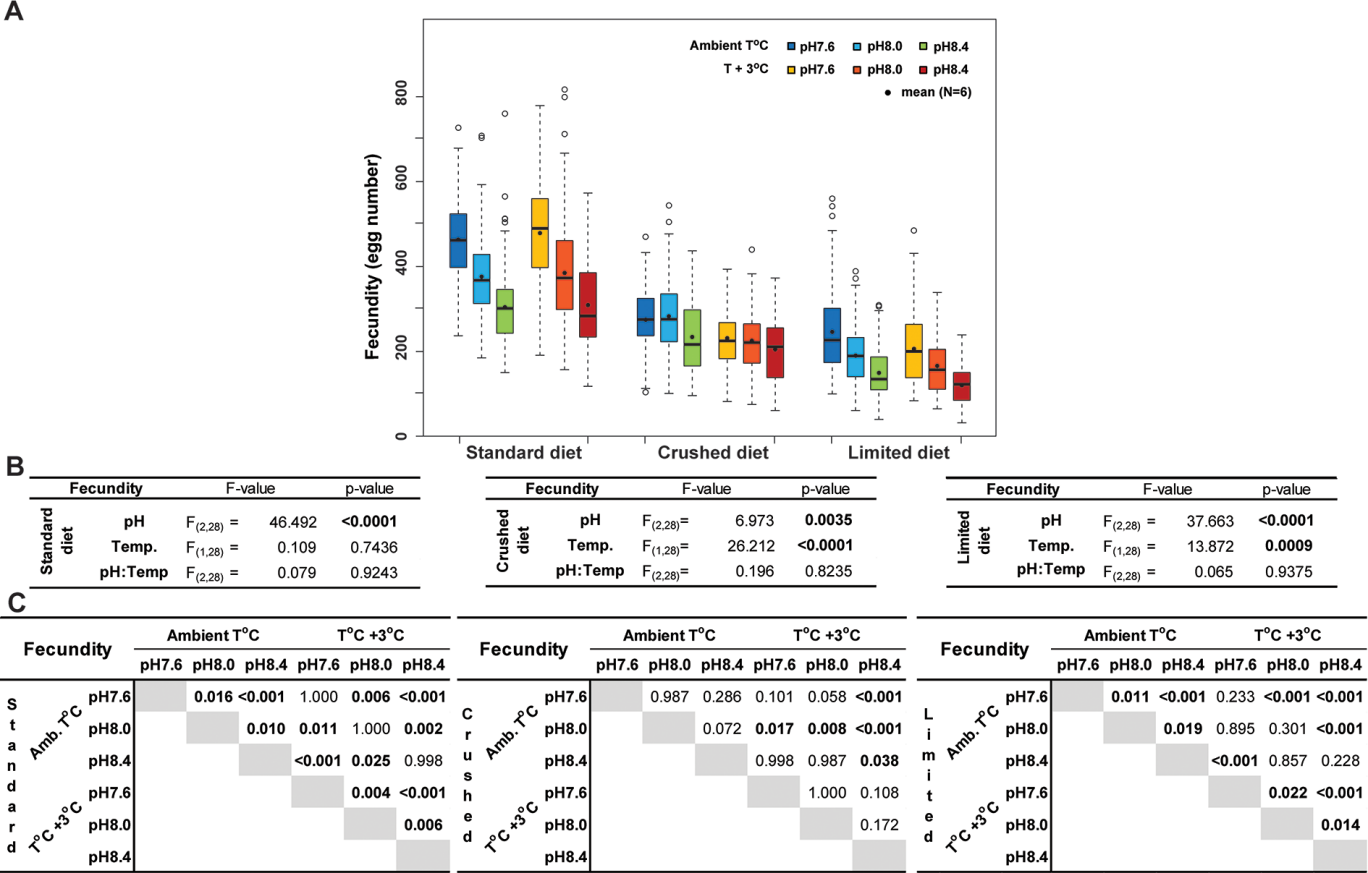
*O*. *dioica* fecundity. (A) Boxplots of *O*. *dioica* fecundity in response to pH and temperature for each diet regime. (B) ANOVA analysis tables. and (C) Tukey test results. Boxplots show the median (thick horizontal black line), the lower and upper quartiles (box) and the whiskers (dashed lines) indicating maxima and minima. When outliers are present (open circles), whisker ends represent the 1.5-fold interquartile range. Means are denoted by filled circles.

Under the standard diet, fecundity (Fig 5) was impacted by pH (F_(2,28)_ = 89.35; p<0.001) but not temperature (F_(1,28)_ = 0.109; p = 0.7436). Females produced increasingly more eggs as pH was reduced from 8.4 to 8.0 to 7.6. Fecundity differences between pH values, at a given temperature, were all significant (Fig 5C), under this regime. The amplitudes of fecundity response between successive pH values were consistent at both temperatures.

Lower fecundity was observed under crushed (mean 36% lower) or limited diets (mean 54% lower) compared to the standard diet. Under these diets, reduced fecundity as a function of pH was even more evident at elevated temperature, where egg numbers were reduced by about 10% compared to the corresponding pH regimes at Amb. T (Fig 5B and 5C). However, there was a difference in the pH-related fecundity response between the limited and crushed diets. Under the limited diet, pH impacted fecundity (F_(2,28)_ = 37.663; p<0.0001; Fig 5B) both within a given temperature regime and between temperature regimes. On the other hand, under the crushed diet, there were no significant differences in fecundity as a function of pH within a given temperature regime. Significant differences were only observed when comparing regimes where both pH and temperature varied (Fig 5C).

After observing differences in fecundity related to pH, temperature and diet, we asked whether gamete quality was also affected by pH and temperature treatments by assessing fertilization rates and juvenile development (Tables 1, 2 and 3). Across all treatments, fertilization rates ranged from 96.0±1.6% (pH7.6/Amb. T) to 97.3±1.9% (pH7.6/+3°C); (pH: F_(2,24)_ = 0.037; p = 0.964; temperature: F_(1,24)_ = 0.837; p = 0.608; Table 2). Juvenile development, quantified 24 h post-fertilization, varied between 64.2±5.9% (pH8.4/T+3°C) and 67.2±6.5% (pH8.0/Amb. T); (pH: F_(2,24)_ = 0.169; p = 0.845; temperature: F_(1,24)_ = 0.608; p = 0.443; Table 2). Thus, based on these developmental parameters, no significant differences were observed in the quality of the gametes produced under any tested treatments.

#### Fitness

Based on fecundity and generation times observed under the standard diet, r_max_ (d^-1^) was calculated as a proxy for fitness (Table 1). Temperature increase, resulted in higher r_max_ values (1.12 to 1.25 at +3°C, compared to 0.90 to 0.99 at Amb. T) (F_(1,17)_ = 664.4; p<0.001; Table 2) by reducing generation time. Moreover, pH reduction was consistently and significantly correlated with superior r_max_ (F_(2,17)_ = 48.256; p<0.001; Tables 2 and 3), an effect mediated by its impact on fecundity (Fig 5). All direct comparisons across both pH and temperature conditions were significant (Table 3).

## Discussion

Warming and oceanic uptake of anthropogenic CO_2_ is altering seawater chemistry with consequences for marine biota [8]. Among species, there will likely be winners and losers, as a result of this acidification [34]. Given a large number of potential interactions, both biotic and abiotic, it is a significant task to understand long term potential impacts of warming and OA on pelagic food webs and marine ecosystems. Accuracy of predictions can be improved with more empirical data combining multiple stressors and observations from single species to the ecosystem level [35]. This work focuses on the appendicularian *O*. *dioica*. Due to their small size and fragility, appendicularians are commonly overlooked, yet there is increasing evidence that they are major contributors to pelagic secondary production, and to vertical carbon cycles in coastal and open ocean waters [16, 36]. Known for rapid blooms with periodic high abundance [20], appendicularians represent a rich food source, with nutritional value equivalent to copepods [21, 22]. Key predators include copepods that feed on appendicularian eggs and juveniles, chaetognaths, ctenophores and various teleost larvae [22]. Therefore, *O*. *dioica* is a relevant species for evaluating the response of pelagic food webs to warmer, acidified oceans.

Over the pH and temperature ranges tested, neither warming nor acidification impacted *O*. *dioica* survival. On the other hand, diet alteration did reduce survival. At elevated temperature, the limited diet reduced survival at all tested pH values. This same effect of elevated temperature was observed when using the crushed diet, but in addition there was a reduction in survival at ambient temperature when pH was elevated to 8.4. The reduced survival in general, at elevated temperature, was further exacerbated at pH 8.4. In mesocosms, the combination of +3°C at pH 7.6 resulted in 2.5- and 5-fold increases in *O*. *dioica* abundance, over the two successive spawning events, when compared to ambient treatment. The temperature effect on developmental progression is well known [28, 37]. Size distributions during the mesocosm experiment illustrated this effect, with earlier reproduction (1–2 days) and faster generation time at elevated temperature, resulting in one additional spawning event over the experiment, relative to ambient temperature. This was also observed in microcosm experiments, though, as expected, the impact of temperature on size was more moderate when animals were cultured under the limited food regime.

Temperature changes are known to affect organismal fitness. All organisms live within a defined range of body temperatures, where the structural and kinetic coordination of molecular, cellular, and systemic processes is optimal [38]. When conditions deviate from this optimal thermal window, there are consequences for survival, interactions with other species, and overall fitness. One driver of organismal performance is oxygen availability. For marine organisms, temperature not only directly impacts dissolved oxygen availability in water, but also affects body fluid circulation, respiration, muscular activity, gas exchange and the onset of protein denaturation, all of which are linked to metabolic activity [39]. The temperature sensitivity of an organism may also vary during the life cycle, as eggs, early larvae and maturing individuals may have narrower temperature range tolerances than juveniles and growing adults [39]. Finally, temperature directly influences geographical distribution, phenology, competition and food web interactions [38, 39].

The effect of temperature on a number of *O*. *dioica*’s physiological parameters has been investigated. When measuring *O*. *dioica* growth rates as a function of temperature, Sato [40] reported a *Q*_*10*_ = 1.68. Using respiration rates measured by Gorsky [41], Lombard et al. [42] calculated *Q*_*10*_ values of 1.77 and 3.03 in the ranges 15 to 20°C and 20 to 24°C, respectively. Similarly, López-Urrutia and Acuña [43], reported a *Q*_*10*_ of 1.96 for respiration, whereas Lombard et al., [42] measured a *Q*_*10*_ value between 15 and 22°C of 2.3. Broms and Tiselius [44] observed a positive correlation between clearance rates and temperature, and calculated a *Q*_*10*_ value of 1.78 between 10–20°C. Finally, López-Urrutia and Acuña [43] reported a *Q*_*10*_ for gut evacuation rate of 1.46 (10–20°C), a value close to 1.4, and considered as an expected value for the respiration rates of poikilotherms ([43], and references therein). Here *Q*_*10*_ values were calculated from both microcosm and mesocosm growth data. Consistent with data from Sato [40], *Q*_*10*_ calculated from microcosm data under standard diet conditions were 1.84 (pH7.6), 1.69 (pH8.0) and 1.64 (pH8.4). Similar values were determined from our mesocosm experiments. When calculating *Q*_*10*_ based on a 3°C temperature increase at a constant pH of 7.6, we obtained a value of 1.70. When the 3°C temperature increase was coupled to a pH decrease from 8.0, to 7.6, the *Q*_*10*_ value was 1.77. Since general metabolic processes are predicted to increase with temperature, feeding processes are also augmented to compensate for higher energy demand [45]. However, although there is an increase in filtration rates at elevated temperatures to compensate for the higher energy demand, diet quality is a factor, with poorer diets resulting in lower *Q*_*10*_ values for growth. Our data suggest a significant reduction in *Q*_*10*_ under suboptimal quality or quantity of food. Under the crushed diet, *Q*_*10*_ averaged 1.33 [1.55 (pH7.6), 1.46 (pH8.0) and 0.97 (pH8.4)], whereas an average of 1.30 was obtained with the limited diet [1.35 (pH7.6), 1.27 (pH8.0) and 1.29 (pH8.4)]. The limited diet was 17% of the standard food regime, and the “crushed” diet was based on lysed algal cells, likely reducing the overall retention of nutrients. Finally, for a given diet and temperature, *Q*_*10*_ values with respect to growth differed between pH conditions. Values calculated at pH7.6 were higher, suggesting that metabolic rates and energetic balance may be improved under acidified conditions, in accordance with our fecundity data, and trends in developmental timing, observed both in mesocosms and microcosms.

**Fig 6:**
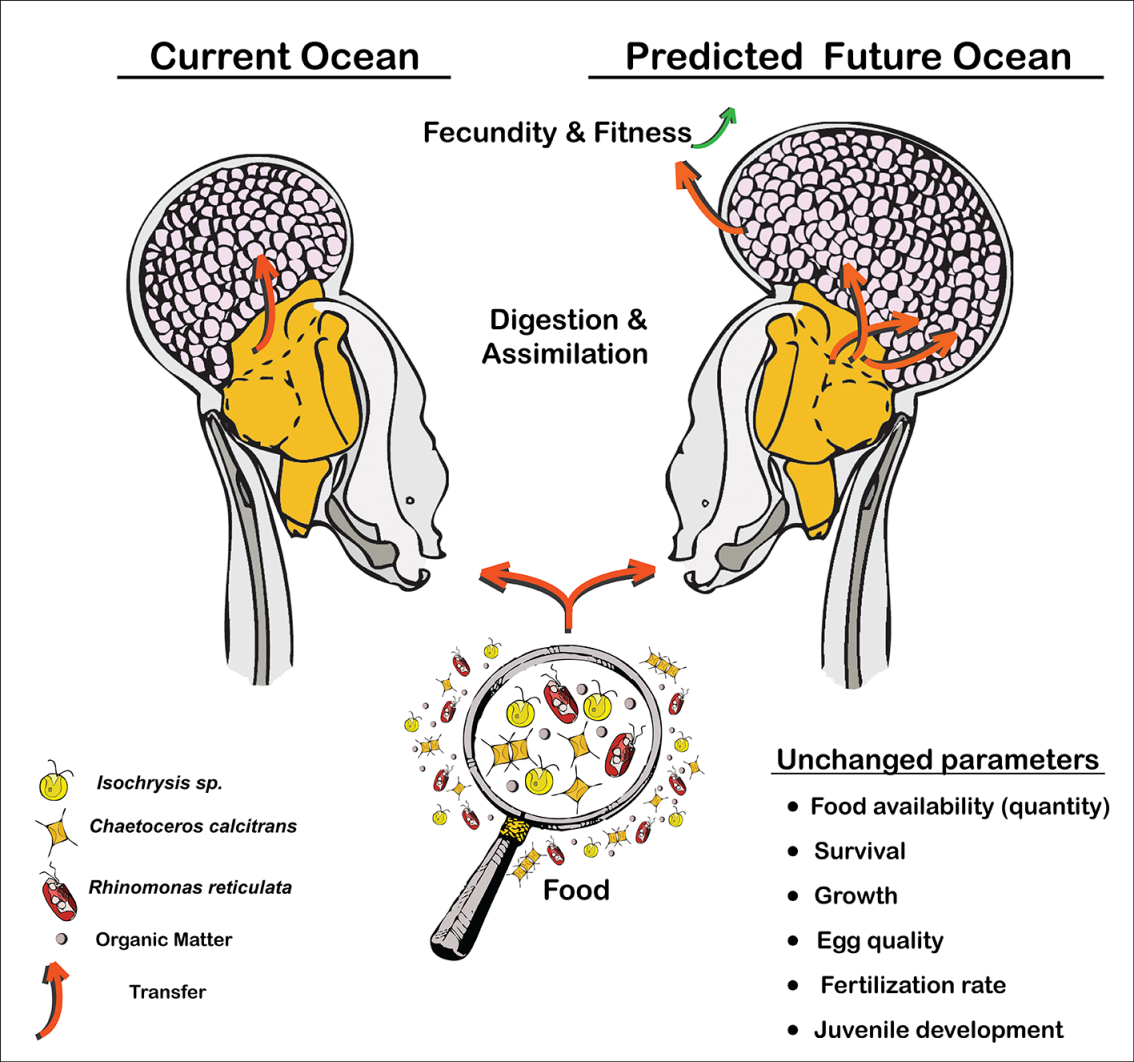
Schematic of the response of *O*. *dioica* to pH variation and warming impacts. Whereas many measured parameters remained unchanged (bottom right), some physiological alterations are manifest in a significant increase of fecundity and therefore population fitness. This may be the result of increased digestion and assimilation efficiency.

In microcosms, at both tested temperatures under the standard diet, fecundity was increased with increasing acidity. Egg production was 20% higher at pH 7.6 compared to pH8.0, whereas it was reduced by 20% between pH8.0 and pH8.4. This agreed with a positive effect of more acidic pH on *O*. *dioica* population dynamics in mesocosms (Fig 6). The physiological homeostasis of marine organisms can be affected by a range of chemical parameters, such as salinity, temperature, dissolved O_2_ and CO_2_ [38], and the maintenance of intracellular pH is essential for many cellular functions that ensure normal development, including calcification, growth, neural function, blood-gas transport, immune responses, behaviour and reproduction [39, 46]. Typically, multicellular organisms have greater passive buffering capacities than unicellular organisms, and many control the pH of their body fluids by secreting or eliminating acid/base through specialized organs [47]. These homeostatic mechanisms permit acclimation to a range of external pH and pCO_2_. However, the ability to compensate the effect of hypercapnia on acid–base balance comes at energetic cost [48], with consequences on metabolic allocation [49, 50]. Less complex marine invertebrates exhibit lower capacity for acid–base regulation [39], and can be more sensitive to alterations in environmental pH [38]. Biological responses to environmental perturbation frequently cause changes in metabolic demand, through changes in biochemical regulation that maintain homeostasis, and ultimately trade-offs in the use of metabolic energy [50, 51, 52]. Therefore, environmental stresses that increase the demand for adenosine triphosphate (ATP) for physiological maintenance may limit the allocation of ATP to growth, reproduction, or locomotion [50, 53]. Our data indicate that *O*. *dioica* will be among organisms able to positively adapt to more acidic conditions anticipated for the world’s oceans.

The activity of digestive enzymes can be affected by external factors, such as temperature, pressure and pH. OA has been shown to cause changes in digestive enzyme efficiency, in mussels and clams [54], echinoderm larvae [55], and flatfish [56]. In pelagic copepods, gut absorption is sensitive to environmental changes, though the function of most digestive enzymes is relatively insensitive to pH changes [56]. One possible interpretation of our data on *O*. *dioica* is that seawater acidification may enhance digestive enzyme activity, and consequently food assimilation. Alternatively, it could favour the acid–base regulatory machinery responsible for stomach pH maintenance. This could result in improved fitness for *O*. *dioica* in response to OA (Fig 6), in contrast to a number of studies that suggest deleterious impacts of OA on animal physiological processes and enzymatic function, including those not directly involved in calcification [57].

The *O*. *dioica* digestive track is composed of various regions, the pharynx, with a short ventral endostyle, the oesophagus, the stomach (left and right gastric lobes), the vertical intestine, the mid-intestine and the rectum [58]. The gastrointestinal pH is neutral to acidic depending on the region, with the left gastric lobe and the mid-intestine being acidic (pH~3) [59]. In these regions, the giant cells of the gastric band contain secretory granules with hydrolytic enzymes (α-amylase, acid phosphatase, non-specific esterase, 5’-nucleotidase, amino-peptidase M). In general, neutral to acidic conditions are characteristic of digestive tracts in chordate larvae [60, 61] and contrast with alkaline pH values reported in the stomach cavities of other marine deuterostomes from the ambulacrarian lineage (echinoderms and hemichordates) [55, 61]. Given the high filtering rates and rapid gut passage times in *O*. *dioica*, a lowered external pH may reduce the energetic cost of maintaining acidic conditions in this “flow-through” digestive tract, thus, enhancing the digestive process.

Previous mesocosm experiments had left open the question as to whether *O*. *dioica* populations performed better at more acidic pH compared to ambient conditions, or instead, more poorly at the more alkaline conditions induced during stimulated algal blooms. The design of the mesocosm experiment here, in concert with a detailed series of microcosm experiments, unequivocally indicate a gradient of increasing fecundity from pH 8.4 to 8.0 to 7.6. When combined with the more rapid generation times at elevated temperature, this indicates that *O*. *dioica* fitness will probably be enhanced under predicted future conditions. The microcosm experiments suggest that part of the explanation may be improved digestion/assimilation efficiency at reduced pH. Both the limited and crushed diets resulted in reduced fecundity at all temperature and pH conditions, indicating nutritional inferiority to the standard diet. However, at both of the tested temperatures, the crushed diet, meant to partially simulate a “pre-digested” food source, abrogated the effect of pH on fecundity, whereas the limited “intact”, reduced food source did not. Despite this leveling effect of the crushed diet on the fecundity response as a function of pH, we did observe increased mortality on the crushed diet at pH8.4, at both temperatures, suggesting that pH8.4 causes additional stress to *O*. *dioica*, beyond reduced digestion/assimilation, which also contributes to reduce overall fitness.

## Conclusions

Our results indicate improved fitness of *O*. *dioica* under predicted warmer and acidified conditions. This arose through shorter generation times at higher temperatures, and increased fecundity at reduced pH, the latter possibly linked to enhanced digestion and assimilation efficiency. Acidification stimulated increases in pico- and nano-plankton [62, 63], bacterial production [64, 65] and POC [66], can also act as bottom-up factors favouring appendicularian abundance [67]. The overall impact of putative altered appendicularian abundances on marine ecosystems remains to be more thoroughly investigated and better defined at the community level, but the data presented here indicate that this gelatinous plankton is likely to be a physiological winner under predicted future scenarios. Thus, despite their fragility with respect to standard sampling protocols, their inclusion in community studies and projected future ocean ecosystem models, will significantly enhance the predictive capacity of such models.

## Supporting information

S1 Table Standard diet regime. (PDF)

S2 Table Mesocosm seawater carbonate chemistry parameters. (PDF)

S3 Table Microcosm seawater carbonate chemistry parameters. (PDF)

S1 Fig Experimental approaches. (PDF)

S2 Fig Physical and chemical parameters in all treatments throughout the mesocosm experiment. (PDF)

S3 Fig *Oikopleura dioica* abundance in mesocosms. (PDF)

S4 Fig Physicochemical parameter monitoring in all treatments and replicates measured during microcosm experiments. (PDF)

S1 Data All datasets from mesocosm experiments. (XLS)

S2 Data All datasets from microcosm experiments. (XLSX)
