# Supplementary material for: Increased fitness of a key appendicularian zooplankton species under warmer, acidified seawater conditions
Source: PLoS One. 2018 Jan 3;13(1):e0190625. doi: 10.1371/journal.pone.0190625 (PMC5752025; doi:10.1371/journal.pone.0190625)
Supplement: S1 Table — (PDF) [file pone.0190625.s001.pdf]

S1 Table. Standard diet regime.

Algal Strains		*Isochrysis sp.*	*Chaetoceros calcitrans*	*Rhinomonas reticulata*	Crushed *Rhinomonas*
**Diet**	***O. dioica* Stages (Day)**	cells.mL^{-1}	cells.mL^{-1}	cells.mL^{-1}	Equivalent fresh cells.mL^{-1}
**Standard**	**0**	2000 / -	2000 / -	- / -	- / -
	**1**	2000 / -	2000 / -	- / -	- / -
	**2**	2000 / -	2000 / -	- / -	- / -
	**3**	2000 / 1000	2000 / 1000	- / 1000	1000 / 1000
	**4**	4000 / 2000	4000 / 2000	1000 / 1000	1000 / 1000
	**5**	4000 / 2000	4000 / 2000	1000 / 1000	1000 / 1000
	**6**	2000 / -	2000 / -	1000 / -	1000 / -

Crushed diet corresponded to algal extracts equivalent to 3 times the number of living cells. Limited diet was defined as 1/6 of the standard diet.
