# Supplementary material for: Increased fitness of a key appendicularian zooplankton species under warmer, acidified seawater conditions
Source: PLoS One. 2018 Jan 3;13(1):e0190625. doi: 10.1371/journal.pone.0190625 (PMC5752025; doi:10.1371/journal.pone.0190625)
Supplement: S2 Table — (PDF) [file pone.0190625.s002.pdf]

S2 Table. Mesocosm seawater carbonate chemistry parameters.

Treatments	Measured				Calculated
	**Temp.** (°C)	**pH** (NBS)	**Salinity**	A_T (µmol kg^{-1})	**pCO_2** (µatm)
T_LP_A:1	12.1 ± 0.7	8.01 ± 0.09	29.3 ± 0.5	2197 ± 44	363.2 ± 63.8
T_LP_A:2	11.8 ± 0.7	8.03 ± 0.11	29.3 ± 0.5	2130 ± 43	338.1 ± 82.5
T_LP_L:1	12.0 ± 0.7	7.72 ± 0.07	29.3 ± 0.5	2194 ± 45	775.6 ± 106.9
T_LP_L:2	12.0 ± 0.7	7.73 ± 0.03	29.3 ± 0.5	2137 ± 63	774.5 ± 49.1
T_HP_L:1	14.4 ± 0.7	7.70 ± 0.05	29.5 ± 0.5	2208 ± 38	851.1 ± 75.0
T_HP_L:2	14.5 ± 0.8	7.69 ± 0.04	29.5 ± 0.4	2158 ± 58	854.4 ± 78.7

Seawater pCO_2 was calculated from pH_{NBS}, salinity and A_T (total alkalinity). All values include mean ± standard deviations from Day 1 to 19. Treatment nomenclature is as defined in Fig.1: T_L, low temperature (the ambient fjord temperature); T_H, high temperature (+3 °C over ambient); P_A, ambient pH (8.0); P_L low pH (7.6), in 2 replicates (:1, and :2).
