# Supplementary material for: Increased fitness of a key appendicularian zooplankton species under warmer, acidified seawater conditions
Source: PLoS One. 2018 Jan 3;13(1):e0190625. doi: 10.1371/journal.pone.0190625 (PMC5752025; doi:10.1371/journal.pone.0190625)
Supplement: S3 Table — (PDF) [file pone.0190625.s003.pdf]

S3 Table. Microcosm seawater carbonate chemistry parameters.

Diet	Treatments pH – Temp.	Measured Temp. (°C)	pH (NBS)	Salinity	A_T (µmol kg^{-1})	Calculated pCO_2 (µatm)
S	pH7.6 - Amb. T	14.3 ± 0.2	7.63 ± 0.03	30.8 ± 1.3	2072 ± 57	1080.5 ± 99.8
	pH8.0 - Amb. T	14.3 ± 0.2	8.04 ± 0.02	30.8 ± 1.2	2103 ± 46	365.8 ± 26.3
	pH8.4 - Amb. T	14.3 ± 0.2	8.45 ± 0.01	30.6 ± 1.2	2393 ± 68	127.1 ± 3.8
	pH7.6 - T. +3°C	17.2 ± 0.2	7.63 ± 0.03	30.7 ± 1.4	2078 ± 57	1210.7 ± 92.8
	pH8.0 - T. +3°C	17.3 ± 0.2	8.05 ± 0.02	30.8 ± 1.3	2093 ± 49	402.3 ± 24.6
	pH8.4 - T. +3°C	17.3 ± 0.2	8.46 ± 0.02	30.6 ± 1.3	2373 ± 51	139.2 ± 9.3
C	pH7.6 - Amb. T	14.2 ± 0.3	7.62 ± 0.02	31.5 ± 0.4	2125 ± 32	1110.6 ± 53.1
	pH8.0 - Amb. T	14.2 ± 0.3	8.03 ± 0.02	31.6 ± 0.4	2108 ± 36	369.1 ± 30.1
	pH8.4 - Amb. T	14.2 ± 0.4	8.45 ± 0.02	31.5 ± 0.5	2395 ± 31	125.8 ± 8.0
	pH7.6 - T. +3°C	17.2 ± 0.2	7.64 ± 0.02	31.5 ± 0.4	2130 ± 36	1203.4 ± 61.1
	pH8.0 - T. +3°C	17.1 ± 0.3	8.02 ± 0.01	31.7 ± 0.4	2119 ± 40	433.8 ± 14.7
	pH8.4 - T. +3°C	17.2 ± 0.3	8.44 ± 0.02	31.5 ± 0.3	2353 ± 24	144.1 ± 7.5
L	pH7.6 - Amb. T	14.2 ± 0.2	7.63 ± 0.03	29.6 ± 0.7	2086 ± 38	1082.1 ± 82.2
	pH8.0 - Amb. T	14.1 ± 0.2	8.05 ± 0.04	29.7 ± 0.7	2082 ± 39	353.3 ± 34.1
	pH8.4 - Amb. T	14.2 ± 0.2	8.45 ± 0.03	29.6 ± 0.7	2346 ± 35	127.7 ± 10.1
	pH7.6 - T. +3°C	17.1 ± 0.2	7.63 ± 0.03	29.6 ± 0.9	2083 ± 40	1208.0 ± 85.6
	pH8.0 - T. +3°C	17.1 ± 0.2	8.04 ± 0.04	29.9 ± 0.8	2085 ± 39	410.9 ± 39.1
	pH8.4 - T. +3°C	17.1 ± 0.2	8.44 ± 0.02	29.5 ± 0.8	2344 ± 38	150.9 ± 9.0

Seawater pCO_2 was calculated from pH_{NBS}, salinity and A_T (total alkalinity). All values show mean ± standard deviations determined at stabilized targeted conditions, from 6 replicates per treatment. Diet: S = standard; C = crushed; L =limited.
