# Supplementary material for: Increased fitness of a key appendicularian zooplankton species under warmer, acidified seawater conditions
Source: PLoS One. 2018 Jan 3;13(1):e0190625. doi: 10.1371/journal.pone.0190625 (PMC5752025; doi:10.1371/journal.pone.0190625)
Supplement: S1 Fig — (PDF) [file pone.0190625.s004.pdf]

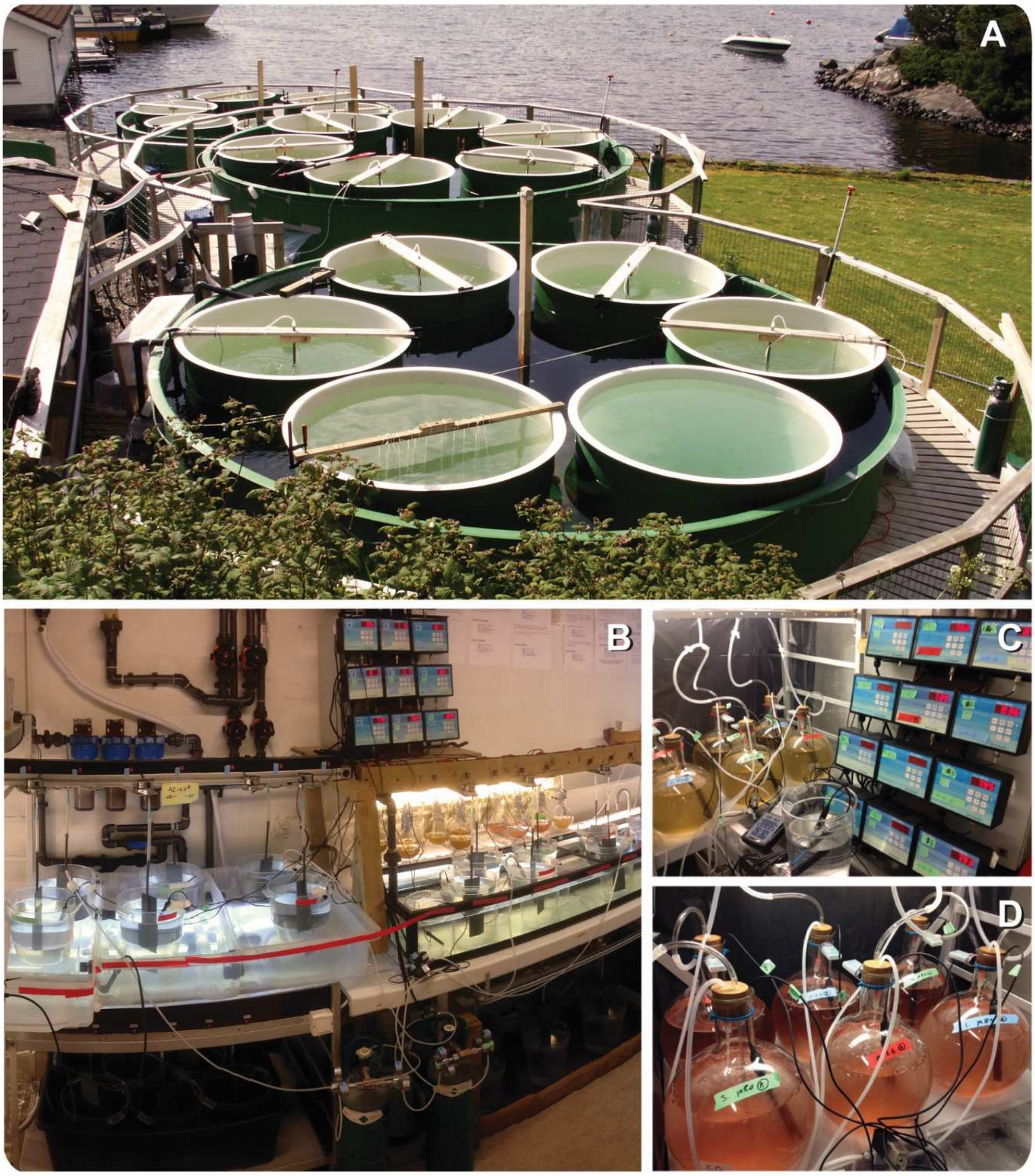

S1 Fig. **Experimental approaches**. A) Mesocosms contained within multiple 2500 L fiberglass tanks placed in larger 25000 L tanks for temperature regulation. B) Microcosms (laboratory scale) using 7 L beakers subject to temperature control in large aquaria. C) pH control system. D) Algal culture in 1 L glass flasks, at room temperature, where pH conditions were manipulated as in (C).
