# Supplementary material for: Increased fitness of a key appendicularian zooplankton species under warmer, acidified seawater conditions
Source: PLoS One. 2018 Jan 3;13(1):e0190625. doi: 10.1371/journal.pone.0190625 (PMC5752025; doi:10.1371/journal.pone.0190625)
Supplement: S2 Fig — (PDF) [file pone.0190625.s005.pdf]

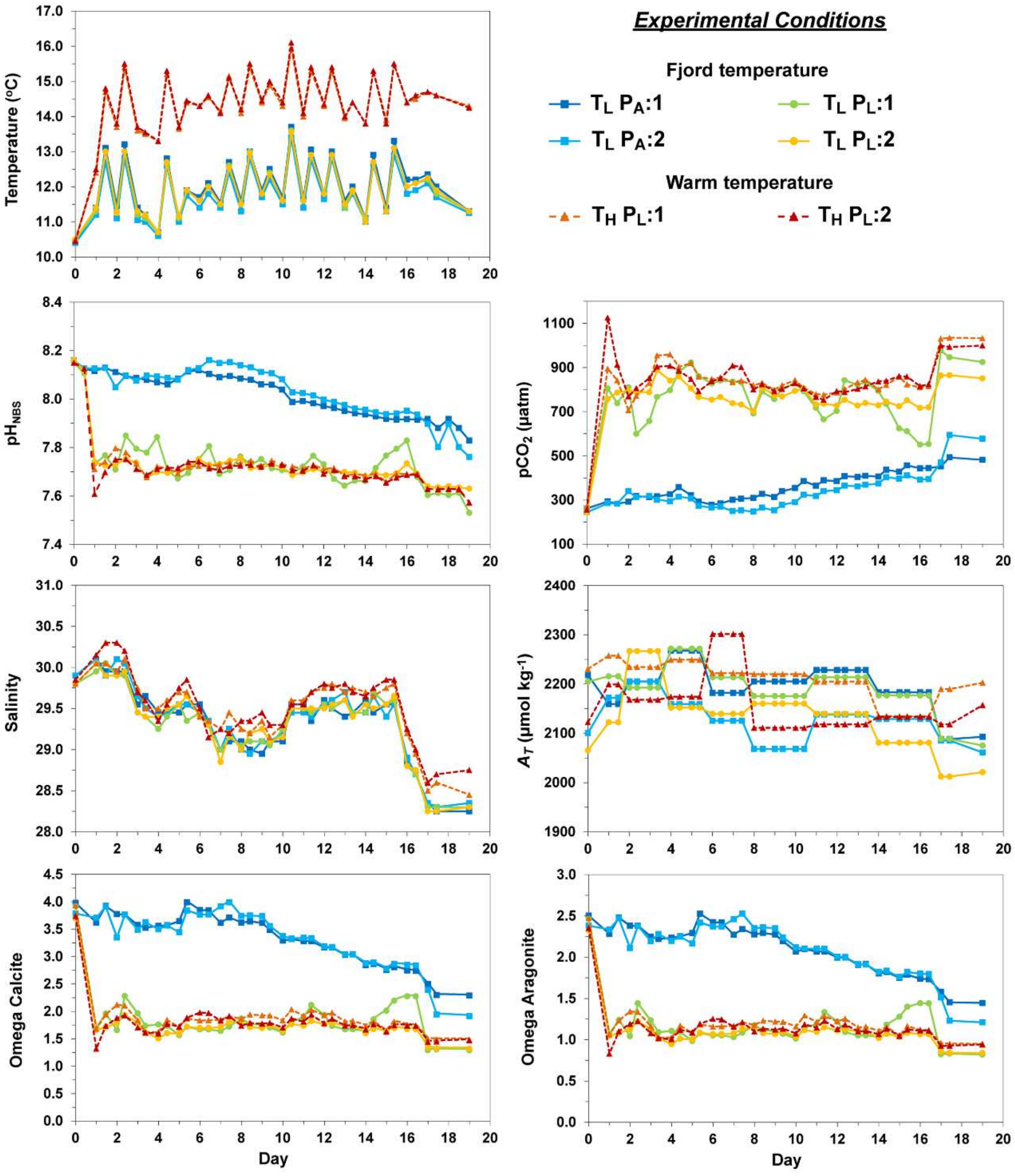

S2 Fig. Physical and chemical parameters in all treatments throughout the mesocosm experiment. Salinity, temperature and pH_{NBS} were measured with a WTW multi-parameter analyzer Multi 3320 equipped with a TetraCon® 925 conductivity probe (301701) and a pH probe Sentix® (103780), calibrated with National Bureau of Standards (NBS) buffers (Hamilton calibration buffer). Total alkalinity (A_T) was determined on filtered samples with a TitroLine Alpha Plus titration system (SI Analytics). pCO_2 was calculated (CO2CALC [29]) from temperature, pH and alkalinity with dissociation constants [30] refitted by Dickson and Millero [31]. Treatment nomenclature is as defined in Fig.1: T_L, low temperature (the ambient fjord temperature); T_H, high temperature (+3 °C over ambient); P_A, ambient pH (8.0); P_L low pH (7.6), in 2 replicates (:1, and :2).
