# Supplementary material for: Increased fitness of a key appendicularian zooplankton species under warmer, acidified seawater conditions
Source: PLoS One. 2018 Jan 3;13(1):e0190625. doi: 10.1371/journal.pone.0190625 (PMC5752025; doi:10.1371/journal.pone.0190625)
Supplement: S3 Fig — (PDF) [file pone.0190625.s006.pdf]

A

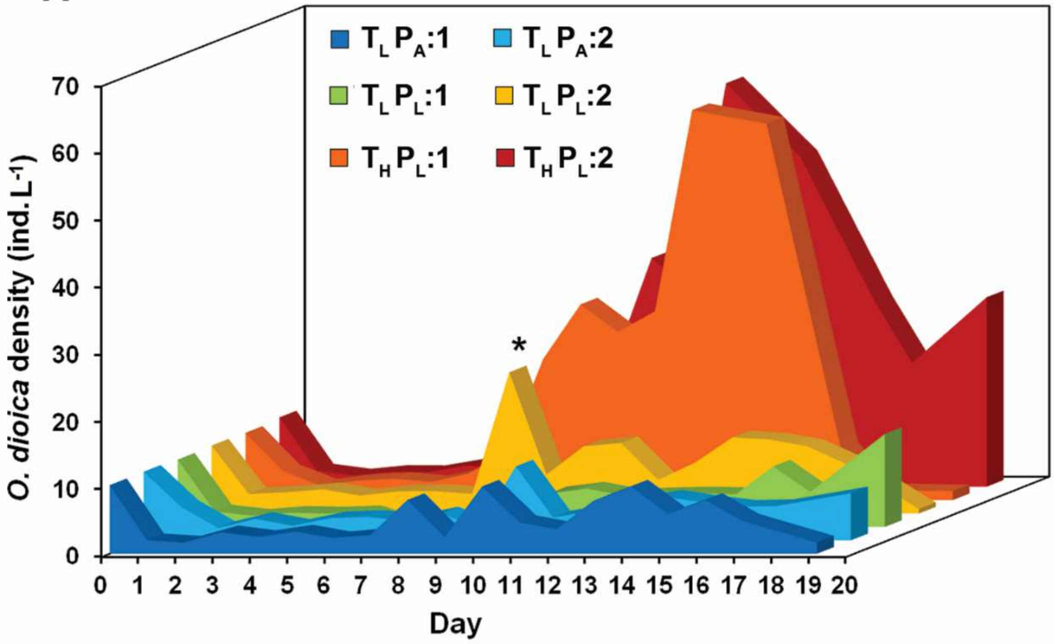

B

	numDF	denDF	F-value	p-value
Days (2-degrees)	2	68	2.86	0.064
pH	1	2	0.76	0.475
Days (2-degrees): pH	2	68	0.26	0.774

C

	**numDF**	**denDF**	**F-value**	**p-value**
Days (3-degrees)	3	66	28.83	**<0.0001**
temp.	1	2	29.49	**0.032**
Days (3-degrees): temp.	3	66	4.22	**0.009**

S3 Fig. *Oikopleura dioica* abundance in mesocosms. A) *O. dioica* abundance in different mesocosm treatments throughout the experiment. B) Results were analyzed as a function of pH at ambient temperature. C) Results were analyzed as a function of temperature under acidified treatment. ANOVA analyses included mesocosm (N=2 per condition) as a random factor and a first-order autoregressive term to account for temporal autocorrelation due to repeated measurement. Polynomial regressions were fitted to the curves, with 2 degrees of polynomial in Days for pH and 3 degrees of polynomial in Days for temperature, defined as the number of spawning events observed for the respective pH and temperature treatments. Significant values are indicated in bold. Treatment nomenclature is as defined in Fig.1: T_L, low temperature (the ambient fjord temperature); T_H, high temperature (+3 ºC over ambient); P_A, ambient pH (8.0); P_L low pH (7.6), in 2 replicates (:1, and :2).
