# Supplementary material for: Increased fitness of a key appendicularian zooplankton species under warmer, acidified seawater conditions
Source: PLoS One. 2018 Jan 3;13(1):e0190625. doi: 10.1371/journal.pone.0190625 (PMC5752025; doi:10.1371/journal.pone.0190625)
Supplement: S4 Fig — (PDF) [file pone.0190625.s007.pdf]

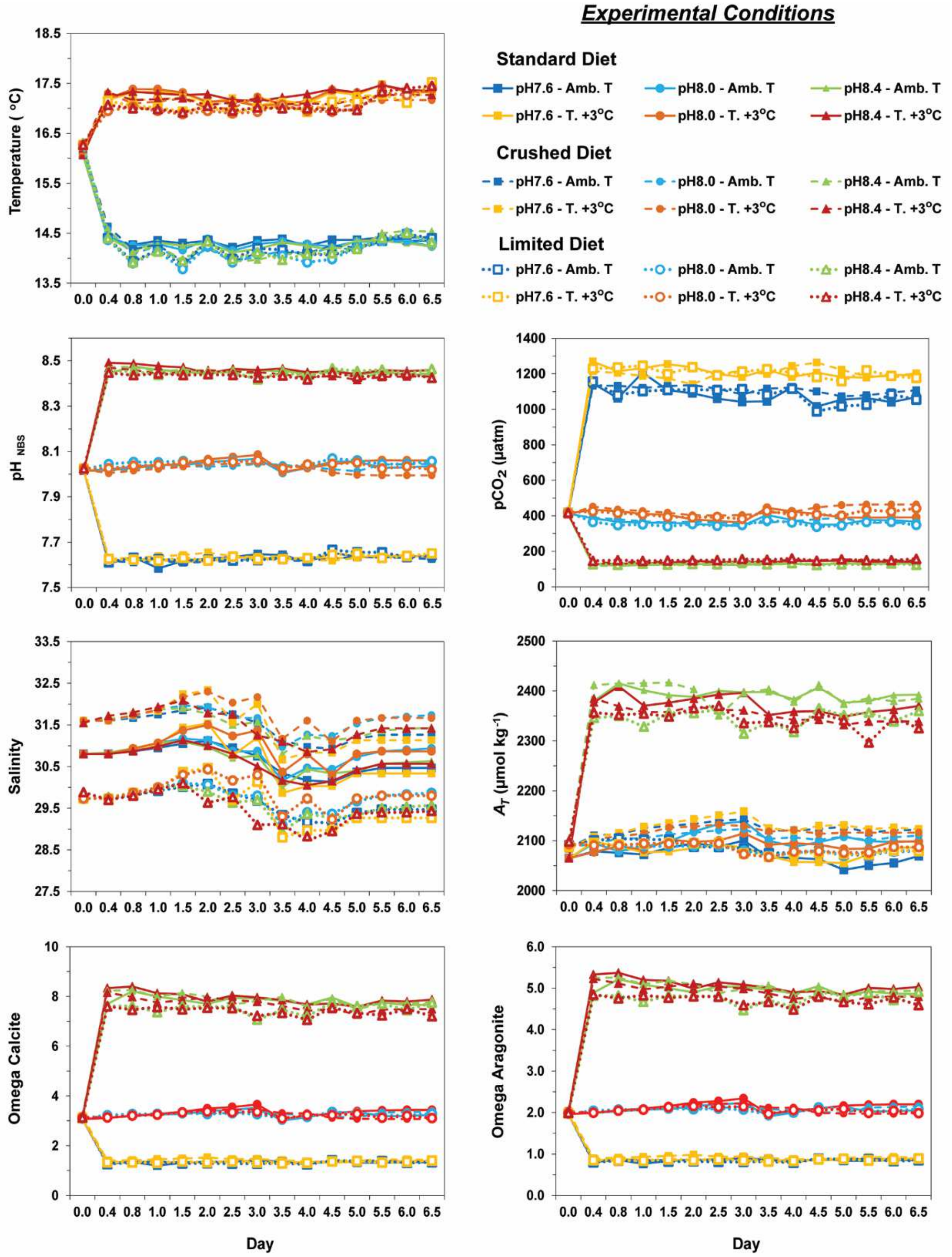

S4 Fig. **Physicochemical parameter monitoring in all treatments and replicates during microcosm experiments**. Salinity, temperature and pH_{NBS} were measured with a WTW multi parameters analyzer model Multi 3320 equipped with a TetraCon® 925 conductivity probe (301701) and a pH probe Sentix® (103780), calibrated with National Bureau of Standards (NBS) buffers (Hamilton calibration buffer). The total alkalinity *(A_T)* was determined on filtered samples with a TitroLine Alpha Plus titration system (SI Analytics). pCO_2 was calculated (CO2CALC [29]) from temperature, pH and alkalinity with dissociation constants [30] refitted by Dickson and Millero [31].
